# Links between Regulatory Systems of ROS and Carbohydrates in Reproductive Development

**DOI:** 10.3390/plants10081652

**Published:** 2021-08-11

**Authors:** Hanako Kiyono, Kazuma Katano, Nobuhiro Suzuki

**Affiliations:** 1 Department of Materials and Life Sciences, Faculty of Science and Technology, Sophia University, 7-1 Kioi-cho, Chiyoda, Tokyo 102-8554, Japan; kiyohana0875@eagle.sophia.ac.jp (H.K.); ktnkzm0519@gmail.com (K.K.); 2 Research Fellow of Japan Society for the Promotion of Science, Chiyoda, Tokyo 102-0083, Japan

**Keywords:** arabinogalactan proteins (AGPs), mitochondria, programmed cell death (PCD), respiratory burst oxidase homolog (RBOH)

## Abstract

To thrive on the earth, highly sophisticated systems to finely control reproductive development have been evolved in plants. In addition, deciphering the mechanisms underlying the reproductive development has been considered as a main research avenue because it leads to the improvement of the crop yields to fulfill the huge demand of foods for the growing world population. Numerous studies revealed the significance of ROS regulatory systems and carbohydrate transports and metabolisms in the regulation of various processes of reproductive development. However, it is poorly understood how these mechanisms function together in reproductive tissues. In this review, we discuss mode of coordination and integration between ROS regulatory systems and carbohydrate transports and metabolisms underlying reproductive development based on the hitherto findings. We then propose three mechanisms as key players that integrate ROS and carbohydrate regulatory systems. These include ROS-dependent programmed cell death (PCD), mitochondrial and respiratory metabolisms as sources of ROS and energy, and functions of arabinogalactan proteins (AGPs). It is likely that these key mechanisms govern the various signals involved in the sequential events required for proper seed production.

## 1. Introduction

To thrive on the earth, highly sophisticated systems to finely control development of male and female gametophytes, pollen–pistil interaction, and fertilization under fluctuating environment have been evolved in plants. In addition, to fulfill the huge demand of foods for the growing world population, it is necessary to understand the mechanisms required for proper seed production in important crops such as cereals and legumes supporting millions of lives in many countries [1]. Thus, dissection of the molecular and physiological mechanisms underlying reproductive development has considered to be a main research avenue to improve the yield of crops.

Despite their toxic potential, reactive oxygen species (ROS) play pivotal roles in the regulation of a broad range of biological processes underlying growth, development, and responses to environmental stresses [2]. In previous studies, contributions of ROS signals to the reproductive development have been revealed [3,4]. Although expression of transcripts encoding ROS producing NADPH oxidases localized in the plasma membrane, RESPIRATORY BURST OXYDASE HOMOLOGs (RBOHs) were shown to be low in reproductive organs a decade ago [5], significance of several RBOHs in the pollen development and pollen tube elongation was then evidenced by the analyses of the mutants deficient in the functions of these enzymes in later studies [6,7,8]. As well as ROS-producing RBOHs, existence of several ROS scavenging systems in reproductive tissues was also reported. Deficiency in these ROS scavenging enzymes resulted in the impairment of reproductive development [9,10,11]. In addition, observations of ROS accumulation in reproductive organs of various plant species demonstrated that ROS distribution altered depending on the stages of reproductive development [12,13,14]. These findings suggest that temporal-spatial patterns of ROS accumulation need to be strictly modulated for the proper reproductive development.

Several lines of evidence indicate the integration of ROS regulatory systems with other signals in the regulation of reproductive development. For example, in Arabidopsis, deficiency in CYCLIC NUCLEOTIDE-GATED CHANNEL 2 (CNGC2) that is known as Ca^2+^ channel resulted in impaired seed production [15] as well as altered ROS accumulation in flowers [16]. These results suggest that modulation of cellular ROS level by the functions of Ca^2+^ channel is essential for seed production. Nitric oxide (NO) was also implicated in reproductive development together with ROS signals. Previous studies demonstrated high level of NO and ROS accumulation in pollen during the hydration on the stigma [17,18], indicating the possible involvement of NO in pollen hydration or germination. Furthermore, NO, a negative chemoattractant of pollen tube guidance, together with ROS, a positive regulator of tip growth, were shown to be required for the pollen tube elongation to the right direction [19,20]. These findings, together with multitasked feature of ROS, Ca^2+^, and NO [2,21,22,23,24], suggest that these signaling molecules might govern the reproductive development by regulating various other pathways.

As sink tissues, reproductive tissues accumulate large amount of carbohydrates during their development. Following the state transition from vegetative stage to reproductive stage, sugars produced in vegetative tissues are transported to reproductive tissues. Proton-based sugar co-transporters, SUCROSE TRANSPOTER (SUC), SUGARS WILL EVETUALLY BE EXPORTED TRANSPOTER (SWEET), and HEXOSE TRANSPORTER (HXT) regulate source-sink sugar transport in a different manner. SUCROSE TRANSPORTERs actively transport sucrose from apoplast to phloem and companion cells by co-transport with H^+^ [25]. In contrast, SWEETs were proposed to release sucrose to phloem cells from neighboring cells through the plasmodesmata [26]. HEXOSE TRANSPORTERs are known as the transporters that uptake monosaccharides such as glucose and fructose at the plasma membrane, as well as co-transports hexose and H^+^ [27]. Some of these sugar transporters are driven by the functions of H^+^-ATPase that might maintain pH in the cytosol and apoplast [25]. In addition, transfer of signals among tissues is important for appropriate distribution of carbohydrates as well as sugar transport throughout the plant. TREHALOSE PHOSPHATE SYNTHASE1 (TPS1), which catalyzes the synthesis of trehalose-6-phosphate (T6P), an intermediate of trehalose synthesis, plays a key role in signal transduction among tissues [28]. Indeed, T6P was shown to detect the sucrose status and convey signals to modulate carbohydrate distribution [29].

A recent study demonstrated that, in petunia, most of genes encoding SUC and SWEET were more strongly expressed in flowers when compared to other organs. Transcript level of these genes increased with flower maturation, reaching to the maximum level when the flower fully opened [30]. Such strong expression of SUCs and SWEETs in flowers indicate the significance of these transporters in the development of reproductive tissues. Indeed, roles of sugar transporters in reproductive development have been revealed in several reports. In Arabidopsis, SUC5 that expresses in developing embryos was shown to be required for the transport of biotin, an essential component for fatty acid synthesis, as well as sucrose from the parent plant to seeds and embryos [31]. In addition, several studies demonstrated that nutrient supply via functions of SWEET5 and SWEET8 is essential for the proper development of pollen [32,33]. Furthermore, different types of sugars were shown to accumulate at different times of floral development. A recent study demonstrated that high level of glucose accumulated in the early stages of the flower but less in the later stages, whereas sucrose accumulated only in mature flowers [34]. Sugar accumulation in reproductive organs at least partially controlled by sugar transporters might be required for the starch synthesis during seed development. Requirement of soluble sugars for the starch synthesis in seeds is also supported by the finding that rice plants exogenously sprayed with sucrose and ABA were significantly improved both in the grain yield and quality via enhanced activities of starch synthesis enzymes in grains [35]. In this process, activity of sucrose transport in the sheath stems was highly improved in response to the ABA plus sucrose treatment. Moreover, several other players consisting of carbohydrates such as arabinogalactan proteins (AGPs) and components of pollen coat as well as metabolisms associated with respiration have been also implicated in the regulation of various processes of reproductive development [36,37,38,39,40] (see sections below).

These findings clearly indicate the importance of the regulatory systems of ROS and carbohydrates in reproductive development. However, it is still poorly understood as to how these regulatory systems function together in reproductive tissues. In this review, therefore, we discuss the mode of coordination and integration between ROS regulatory systems and carbohydrate transports and metabolisms that are required for the reproductive development. In particular, mechanisms involved in the development of male and female gametophytes, pollen adhesion and hydration, pollen germination and pollen tube elongation, and perception of pollen tube tip by female gametophyte are discussed. However, we do not cover all aspects of regulatory systems of ROS and carbohydrates that function in reproductive tissues. For more details on the mechanisms underlying reproductive development, we would like to refer our readers to more extensive reviews (see [3,4,41,42,43,44]).

## 2. Development of Male Gametophytes

As the initial step of the development, anthers form a single layer of archesporial cells, which is divided into sporulation tissues and parietal cell layer via periclinal division. Sporulation tissues are then developed into pollen mother cell directly or by splitting. Pollen mother cells produce four microspores (pollen tetrad) that undergo meiosis followed by somatic cell division to produce pollen tube cell and generative cell [45]. The parietal cell layer constitutes of the layers in order from the outside: epidermis, endothelium, middle layer, and tapetum, and encloses sporulation tissue [46].

Previous studies demonstrated the significance of ROS and redox regulatory systems in the development of anthers as well as pollen. For example, in Arabidopsis, two functionally redundant GLUTALEDOXINs (GRXs), ROXY1 and ROXY2, were shown to play essential roles in the early steps of tapetum differentiation [11,18,47]. *roxy1/roxy2* double mutants exhibited abnormal patterns of tapetum differentiation as well as failure of pollen production [11,47]. Hong and co-workers also showed that a rice mutant deficient in MICROSPORELESS1 gene (*mil1*), encoding a GRX, did not produce microspores in anthers. Anthers of *mil1* plants were defective in the meiotic entry of cells in sporulation tissues and failed the differentiation of surrounding parietal cell layer [9]. These findings suggest that GRXs are essential for the differentiation of tapetum, leading to the proper development of pollen. Tapetum is in contact with microspores or pollen grains during their formation and plays an important role in supplying nutrients and materials. During the later stages of the maturation, pollen is separated from the anther wall, and the nutrients are absorbed from the anther solution that fills the anther chamber. SUGARS WILL EVETUALLY BE EXPORTED TRANSPOTER 8 (SWEET8) in tapetum was implicated in the release of glucose from the anther wall into the anther chamber [26,32]. This glucose release via the function of SWEET8 might be significant for the pollen wall formation as well as maintenance of pollen sterility [32]. In addition, sugar supply to developing pollen is required for the synthesis of starch, a key determinant of pollen viability [41]. The synthesis of starch depends on the availability of sucrose and hexose, as well as the capacity of microspore to utilize these sugar sources. In previous studies, monosaccharide symporters, SUGAR TRANSPORTER 6 (STP6), and SUC1 were shown to be important for acquisition of sucrose and hexose by microspore [48,49]. Furthermore, accumulation of sucrose itself during the later stage of pollen maturation might be also essential for the maintenance of desiccation tolerance of pollen [41,50]. These findings suggest that functions of GRXs and sugar transporters need to be strictly coordinated for the development of pollen. Proper differentiation and development of tapetum might be required for its function to supply nutrients to developing pollen. Thus, it should be interesting to investigate how activity of SWEET8 is regulated by GRX-dependent redox signals. Furthermore, it is also necessary to elucidate how coordination among different sugar transporters are controlled.

Degradation of tapetum via programmed cell death (PCD) in the proper timing is also essential for pollen maturation and achievement of fertilization by nourishing microspores [51]. The expression of genes that are involved in various developmental processes in tapetum, such as ETERNAL TAPETUM 1, a basic helix-loop-helix transcription factor that promotes PCD, reach to the maximum level during meiosis of pollen mother cells, supporting the idea that, at the time of microspore formation, tapetum degradation occurs due to PCD [52]. In Arabidopsis, a ROS-producing NADPH oxidase, RBOHE was found to be expressed in the tapetum and to positively regulate PCD of tapetum cells. Arabidopsis mutants deficient in RBOHE exhibited impaired pollen development accompanied by reduced ROS production and delayed tapetum PCD [6]. Contrarily, overexpression of RBOHE resulted in increased ROS and early tapetal degradation that caused impaired pollen development [6]. These results suggest that timing and amount of RBOH-dependent ROS production need to be fine-tuned for the regulation of tapetum degradation and pollen development. In addition, the role of arabinogalactan protein 2 (AGP2), a family of heavily glycosylated proteins [53], in the regulation of RBOH-dependent ROS production was demonstrated in a recent study. Knockdown of AGP2 in rice resulted in enhanced expression of genes encoding RBOHs, increased level of ROS, and abnormal anther development accompanied by premature initiation of PCD and pollen abortion [40]. Many of the AGP family members possess a glycosylphosphatidylinositol (GPI) lipid anchor that allows them an association with the plasma membrane [53]. Thus, AGPs might be involved in the perception and transduction of signals that control various biological processes [54]. Although further studies are still required, it seems to be reasonable that AGPs and RBOHs localized in the plasma membrane function together to fine-tune the timing of tapetum PCD.

In Arabidopsis, PEROXIDASE9 (PRX9) and PRX40 play key roles in the maintenance of microspore cell wall integrity [10]. PRX9 and PRX40 were shown to crosslink extensins in the cell wall of microspore. *prx9/prx40* double mutants exhibited impaired pollen development accompanied by deficiency in extensin cross-linking and compromised cell wall integrity [10]. Sun and co-workers demonstrated phenotypic defects of Arabidopsis mutant deficient in SWEET8 (named “*ruptured pollen grain 1*”, *rpg1*) at early stages of primexine (early exine) formation in microspore [55]. In this mutant, callose wall deposition as well as CALLOSE SYNTHASE 5 expression were reduced. More detailed observation of microspores by electron microscopy revealed aberrant primexine formation at the tetrad stage in *rpg1* mutant. In addition, by this abnormality in primexine formation, the accumulation of sporopollenin, the main component of exine, was disturbed in *rpg1* mutant [55]. Involvement of both SWEET8 and PRX-dependent mechanisms indicate the possible integration between sugar transport and ROS regulatory systems in the formation of microspore cell wall. Further studies are still required to clarify the mode of their integration.

After the maturation of pollen, alterations in the feature of endodermis are essential for the proper timing of anther cleavage to release pollen. When the endothelium is wooded by lignin, organic polymers synthesized by cross-linking phenolic precursors, the endodermis cells at the anther cleavage site are subjected to chemical stimuli. Endodermis turgor pressure then increases and the bonds between cells are broken, resulting in the anther cleavage [56]. A transcription factor MYB26 was shown to function upstream of the lignin biosynthetic pathway and plays a regulatory role in endothelial wood formation, as evidenced by the absence of wall thickening observed in endothelial cells of mutants lacking MYB26 [57]. In addition, the importance of jasmonic acid (JA) and auxin have been also reported in anther cleavage. JA promotes dehydration of endothelium and storm cells, leading to bond breaking between cells [58]. On the other hand, auxin negatively regulates the cleavage of anthers by regulating JA signals [59]. Furthermore, the chloroplast-localized cystathionine β-synthase X 1 (CBSX1) that interacts with and activates THIOREDOXINs (TRXs) was implicated in anther dehiscence [18]. Overexpression of CBSX1 or CBSX2 in Arabidopsis resulted in severe sterility caused by inhibition of anther dehiscence with decrease in H_2_O_2_ that caused lignin deficiency [18]. It is likely that the redox regulation involving CBSX1 and TRXs might modulate H_2_O_2_ level. Unfortunately, it is still not clear as to how ROS regulatory systems function to control anther cleavage in detail. However, we can speculate the integration between ROS and hormone signals because such integrations in various biological processes have been reported in numerous studies [4,60,61].

Taken together, these findings indicate that proper development of anthers and pollen is dependent on temporal-spatial coordination of ROS regulatory systems and carbohydrate transports and metabolisms. In particular, integration of signals involving ROS and carbohydrates should be strictly controlled in the tapetum formation and PCD. Reactive oxygen species signals modulated by the balance between producing and scavenging systems might be a key to govern the various mechanisms. In addition, AGPs, a modulator of RBOHs activity, might play key roles to link ROS production and sugar status in cells. Interestingly, AGP2 in rice was shown to downregulate expression of gene encoding HEXOKINASE (HXK) by modulating the DNA methylation in the HXK promoter region [40]. HEXOKINASE is known as sensor of sucrose, monitoring the energy status in the cells [62]. Furthermore, SWEET8 that could function with GRXs and PRXs might be also a key player to link ROS regulatory systems and sugar transport.

## 3. Development of Female Gametophytes

To achieve fertilization, proper development of female gametophytes, as well as male gametophytes, is also important. In previous studies, unique pattern of O_2_^−^ elaborate distribution was detected in the mature female gametophytes. Although O_2_^−^ accumulated in the central cell of the female gametophytes, it was absent from the micropylar cells [63,64]. Analyses of *oiwa* mutant that is deficient in mitochondrial MANGANASE-SUPEROXIDE DISMUTASE (MSD1) revealed that mitochondrial ROS regulatory system is essential for the determination of cell fate and embryo sac polarity [63,64]. In *oiwa* mutant, accumulation of ROS was detected in the micropylar cells as well as the central cell. The high level of mitochondrial superoxide in this mutant might lead to the abnormal phenotypes of female gametophytes such as arrest of mitosis during megagametogenesis and impaired specification of egg cells [63,64]. Tetrapyrrole biosynthesis was also proposed as another key process to modulate mitochondrial ROS production required for the development of female gametophytes. *hemn1* mutant in Arabidopsis that is deficient in tetrapyrrole biosynthesis exhibited defects in the viability of pollen and embryo sacs accompanied by unfused polar nuclei [65]. Central cell differentiation was also impaired in *hemm1* mutant, resulting in abnormal development of endosperm and embryo. In addition, involvement of AGPs in the development of female gametophyte was also speculated from hitherto studies. For example, AGP18 was shown to be expressed specifically in the megaspore mother cell. Specific glycosylation pattern of AGP18 was found only in the functional megaspore, indicating the significance of AGP18 in the determination of cell fate [66,67].

Although key factors mentioned above (i.e., MSD1, HEMM1, and AGP18) have been found in previous studies, molecular mechanisms underlying development of female gametophytes are poorly understood compared to those underlying male gametophytes. Further studies are required to dissect key mechanisms that function in different parts or at different timing.

## 4. Pollen Adhesion and Hydration

Following the development of male and female gametophytes, sophisticated interactions between pollen and pistil initiate with the landing of pollen on the stigma. It is easy to imagine that characteristics of pollen itself are determinant for the efficiency of adhesion and hydration of pollen. Concentration of osmoticants such as sucrose and hexose in pollen was shown to be important for the adhesion and hydration of pollen to the stigma [68]. Indeed, water-absorbing capacity of pollenkitt might be a key for the adhesion and hydration of pollen to the stigma [68]. Together with the findings discussed in the section above (see “Development of male gametophytes”), we can speculate that the activity of sugar transporters controlled by ROS and redox signals during pollen development could contribute at least partially to the accumulation of osmoticants required for pollen adhesion and hydration to the stigma. In addition, involvement of pollen ROS in the modulation of physical property of pollen walls was proposed in previous studies. Scission of polysaccharides via the function of OH− results in loosing of pollen wall, probably leading to the acceleration of pollen hydration [69]. In contrast, H_2_O_2_ strengthens polymers via peroxidase-mediated cross-linking of hydroxycinnamates [44,70]. In Arabidopsis, KINβγ is known as a subunit of SUCROSE NON-FERMENTING 1-RELATED PROTEIN KINASE 1 (SnRK1) complex that is essential for maintenance of the functions of the mitochondria and peroxisomes [71]. Interestingly, deficiency in KINβγ resulted in the reduced ROS level in the pollen grains accompanied by compromised pollen hydration [71]. Furthermore, structure of mitochondria and peroxisomes were destroyed in KINβγ-deficient plants. As mitochondria and peroxisomes are main sources of ROS generation in the cells [2], we can hypothesize that strict modulation of ROS level generated from these organelles in pollen might be essential for the pollen hydration.

Several studies revealed the detailed dynamics of ROS and NO accumulation in reproductive organs [13,18,72,73]. Prior to the landing of pollens on the stigma, high level of NO and H_2_O_2_ accumulate in pollens and papilla cells, respectively. After the landing of pollen grains on the stigma, NO derived from pollens might function to decrease the level of H_2_O_2_ accumulation in papilla cells [17,18,73]. Indeed, it was reported that NO activates several ROS scavenging systems [74]. In addition, a recent study demonstrated increase in Ca^2+^ level in papilla cells after the landing of pollen on the stigma [13]. Decrease in ROS accumulation in the stigma by pollen attachment might be a unique phenomenon for the reproductive tissues, because ROS producing enzyme, RBOHs are well known to be activated by Ca^2+^ [5]. Alterations in the accumulation of these signaling molecules in reproductive organs indicate the dramatic changes in the signals before and after pollen attachment to the stigma.

High accumulation of ROS in the stigma prior to the pollen attachment was proposed to be essential for pollen–pistil interaction, as well as defense mechanisms against microbe attack [17,44,75]. Decreased level of ROS in the stigma by the exogenous application of flavonoids inhibited the attachment of pollen to the stigma [76], suggesting the significance of high level of ROS accumulation in the stigma for pollen adhesion. Although high level of ROS in the stigma before pollen attachment is essential for the pollen adhesion [76], decrease in level of ROS in the stigma after the pollen attachment is important for the pollen hydration. A recent study demonstrated that perception of POLLEN COAT PROTEIN-Bs (PCP-Bs) by ANJEA-FERONIA (ANJ-FER) receptor kinase complex is required for pollen hydration [14]. Extracellular domain of FER was shown to directly interact with PCP-Bs. In addition, RAPID ALKALINIZATION FACTORS 23 and 33 (RALF23 and 33) also directly interact with ANJ-FER and enhance production of ROS in stigma via function of RBOHD before pollen attachment. PCP-Bs inhibit interaction of RALFs and ANJ-FER, and compromises ROS production when pollen lands on stigma.

The plasma membrane of the stigmatic papilla cells releases secretion that contains enzymes such as cellulase and pectinase. These enzymes then promote hydration of pollen by degrading the pollen coat [77]. In addition, the accumulation of cellulase increases in pollen as pollen maturation progresses [78]. In Arabidopsis, EXOCYST SUBUNIT EXO70 FAMILY PROTEIN A1 (EXO70A1), an exocyst complex subunit in stigma, was shown to be required for the pollen hydration [79]. EXOCYST SUBUNIT EXO70 FAMILY PROTEIN A1 was proposed to function in secretion of stigmatic cells to deliver the vesicles containing aquaporins that increase water permeability, leading to acceleration of pollen hydration [79]. Phosphatidylinositol-4-phosphate (PI4P) was also known to be essential for the initial process of pollen–pistil interactions. Arabidopsis plant deficient both in PHOSPHATIDYLINOSITOL-4-KINASE β1 and β2 (PI4Kβ1 and β2) demonstrated the compromised pollen grain hydration accompanied by lower PI4P [37]. Interestingly, interaction of EXO70A1 with PI4P was indicated in animals and yeast [80,81]. Thus, it should be important to test if EXO70A1 in plant interact with lipids to regulate pollen hydration on the stigma.

**Figure 1:**
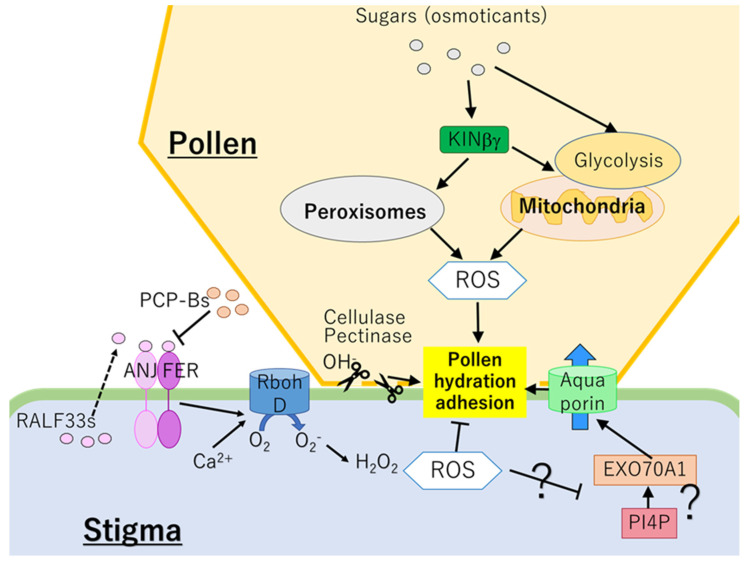
Mechanisms regulating pollen hydration and adhesion. In pollen, KINβγ might modulate ROS level and carbohydrate homeostasis depending on the sugar status via controlling the functions of mitochondria and peroxisomes. ROS derived from the mitochondria and peroxisomes in pollen are required for the pollen adhesion and hydration. In contrast, decrease of ROS in stigma might be important for the pollen adhesion and hydration, suggesting that ROS in stigma could negatively regulate these processes. Binding of RALF33 to ANJ-FER activates RBOHD-dependent ROS production. In contrast, PCP-Bs derived from pollen compete with RALF33s for the binding to ANJ-FER and inhibit RBOH-dependent ROS production. Effects of ROS on EXO70A1 or PIP4 need to be analyzed in future studies. ANJ: ANJEA, EXO70A1: EXOCYST SUBUNIT EXO70 FAMILY PROTEIN A1, FER: FERONIA, PCP-Bs: POLLEN COAT PROTEIN-Bs, PI4P: phosphatidylinositol-4-phosphate, RALF33: RAPID ALKALINIZATION FACTOR 33, ROS: reactive oxygen species.

Although direct evidence has not been provided, the cross-talks between ROS signals and carbohydrate metabolisms during the pollen adhesion and hydration can be speculated (Figure 1). SUCROSE NON-FERMENTING 1-RELATED PROTEIN KINASE 1 (SnRK1) in pollen might be one of key players to mediate ROS regulatory systems and carbohydrate metabolisms. Not only as ROS generator, SnRK1 is well known as a hub to switch energy metabolisms depending on the surrounding sugar status [82]. It is therefore necessary to elucidate how ROS regulatory systems and carbohydrate metabolisms are integrated via the functions of SnRK1 to regulate pollen adhesion and hydration. One possibility is that SnRK1 might accelerate ROS production by activating glycolytic pathway that provides NADH to the mitochondrial electron transport chain. Indeed, it has been demonstrated that SnRK1 can activates glycolysis and authophagy to obtain energy by degrading carbohydrates with high molecular weight [82,83,84]. Furthermore, it is also necessary to elucidate how decrease of ROS in the stigma via the function of PCP-Bc and scavenging systems promote pollen hydration. It is likely that effects of ROS on the activity of EXO70A1 or amount of PI4P need to be analyzed in future studies.

## 5. Pollen Germination and Pollen Tube Elongation

Following the hydration, pollen germinates, and the pollen tubes penetrate into the stigma. Pollen tubes then pass the style tissue that connects the stigma and the ovary, transmitting tract (TT), and finally reach to the ovule [85].

**Figure 2:**
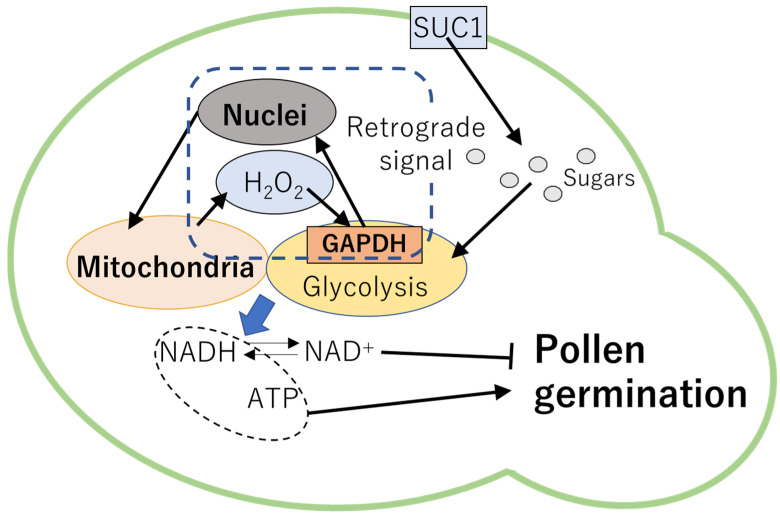
Mechanisms regulating pollen germination. SUC1 might supply sugar sources metabolized in glycolysis. ROS generated from the mitochondria might be perceived by GAPDH, a key enzyme in glycolysis. GAPDH might mediate ROS-dependent retrograde signaling that regulate metabolisms to generate energy required for pollen germination. NADH/NAD^+^ ratio can also affect pollen germination. NAD^+^ accumulated in pollen might inhibit the metabolic processes that are required for pollen germination. GAPDH: GLYCERALDEHYDE-3-PHOSPHATE DEHYDROGENASE.

In previous studies, significance of carbohydrate transport and metabolisms in the regulation of pollen germination has been demonstrated. For example, Arabidopsis mutant deficient in SUC1 exhibited impaired pollen development and low rates of pollen germination in vitro [86]. In lily, respiration rate rapidly increased in pollen prior to emergence of pollen tube [38], suggesting that reprograming of the respiratory metabolisms could be required for the pollen germination. Indeed, glycolysis was shown to be an important pathway to generate energy (i.e., ATP and NADH) for pollen germination as well as pollen tube elongation [38]. In addition, plants might possess mechanisms to modulate metabolisms depending on the redox states in cells. It has been demonstrated that the timing of pollen germination is dependent on the accumulation of NAD^+^ during pollen maturation [87]. Generation of ROS via respiration as well as pollen germination were inhibited when NADH/NAD^+^ ratio was low. It is likely that NAD^+^ accumulated in pollen might inhibit the metabolic processes that are required for pollen germination [87]. Furthermore, GLYCERALDEHYDE-3-PHOSPHATE DEHYDROGENASE (GAPDH) in the cytosol might function as sensor of H_2_O_2_ diffused from the ROS-producing organelles [38] when imbalance of electron transport chains occurs. Function of GAPDH as H_2_O_2_ sensor might be important to regulate signals from organelles to nuclei, called retrograde signaling [88,89,90,91]. These findings suggest that ROS-dependent retrograde signaling might be a key mechanism to modulate metabolisms required for pollen germination depending on the energy states in cells (Figure 2). In addition, redox regulation of glycolysis might be also essential to mediate ROS and sugar homeostasis.

**Figure 3:**
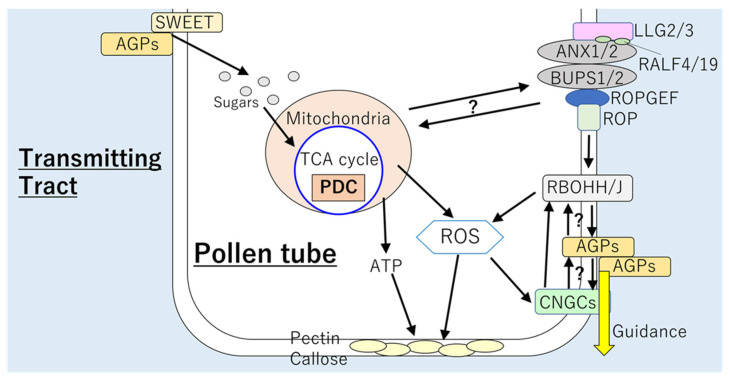
Mechanisms regulating pollen tube elongation. Sugars are provided from transmitting tract via functions of SWEET or AGPs. Then, sugars in pollen tube might be utilized in the respiratory metabolisms that generate ROS and ATP. PDC might play key role to provide energy required for pollen tube elongation. RBOH-dependent ROS were shown to be regulated by ANX1/2–BUPS1/2–LLG2/3 receptor complex. This receptor complex activates RBOHH/J-dependent ROS production via functions of GEF and ROP. ROS produced by respiration and RBOHs might be important for pectin and callose deposition at the tip wall of the pollen tube. In addition, RBOH-dependent ROS signals could be integrated with signals associated CNGCs and AGPs to regulate direction of pollen tube growth. However, links between ROS-CNGC signals and AGPs are still poorly understood. AGPs: arabinogalactan proteins, ANX1/2: ANXUR1/2, BUPS1/2: BUDDHA’S PAPER SEAL 1/2, CNGC: CYCLIC NUCLEOTIDE GATED CHANNEL, GEF: GUANINE NUCLEOTIDE EXCHANGE FACTOR, LLG2/3: LORELEI-LIKE-GPI-ANCHORED PROTEIN 2/3, PDC: PYRUVATE DECARBOXYLASE, RALF4/9: RAPID ALKALINIZATION FACTOR 4/9, RBOHH/J: RESPIRATORY BURST OXIDASE HOMOLOG H/J, ROP: RHO of PLANTS, ROS: reactive oxygen species.

Mechanisms that constitute of various players regulating pollen tube elongation has been extensively studied (Figure 3). The TT, pass of pollen tube, consists of cylindrical cells filled with large amount of fibrous extracellular matrix (ECM). The space for pollen tube elongation in the TT is known to be formed by PCD of cells in the septum [92,93]. In tobacco and petunia, STIGMA SPECIFIC PROTEIN 1 (STIG1), expressed in pistil tissues, is known to promote pollen tube elongation via accelerating ROS production as well as secretion of the extracellular matrix [94,95]. In addition, GRIM REAPER (GRI), a STIG1 ortholog in Arabidopsis, was also shown to promote O_2_^−^ production that triggers cell death [96]. These facts suggest that STIG1 and GR1 might play key roles to accelerate ROS-induced PCD in the TT. Extracellular matrix in the TT is rich in polysaccharides, glycolipids, and glycoproteins. These compounds in the ECM are utilized as energy supply that attracts the pollen tube elongation [97]. For example, it was proposed that when TT is incorporated into the pollen tube wall, an arabinogalactan protein, TRANSMITTING TRACT SPECIFIC (TTS), is deglycosylated and the energy sources for pollen tube elongation, arabinogalactans, are released [98]. Furthermore, several studies also revealed the significance of sugar transporters in the promotion of pollen tube elongation. A recent study demonstrated that sugar transporter protein MdSTP13a in apple uptakes sucrose for pollen tube elongation. Sorbitol, a major photosynthate in apple, might control MdMYB39L that binds to promoter of MdSTP13a to activate its expression [99]. In addition, expression of SWEET5 in Arabidopsis was observed in germinated pollen and it might supply sugar to pollen grain vegetative cells in late pollen development [33]. In future studies, it is necessary to understand how these sugar transporters function together with mechanisms to release energy sources from the TT.

It was reported that respiration rate during the pollen tube elongation increases to almost twice as much higher than that during pollen germination [38]. In pollen tube, mitochondria can be observed proximity to the subapical area. This localization of mitochondria might be associated with the requirement of ATP for the pollen tube elongation [38]. Nevertheless, the electron transport chain might not be a main source of energy. Previous studies demonstrated that metabolic pathways are strictly modulated to support the energy generation via respiration. PYRUVATE DECARBOXYLASE (PDC) might play important roles to support the functions of TCA cycle, which can generate ATP. PYRUVATE DECARBOXYLASE converts pyruvate to acetaldehyde, which is oxidized to acetate. Then, acetate is converted to Acetyl-CoA, which is utilized in the TCA cycle [100,101,102]. Significance of this pathway involving PDC was supported by the fact that Arabidopsis deficient in PDC2 exhibited impaired pollen tube elongation through the style [102].

Interestingly, accumulation of ROS in pollen tube coincided with the localization of mitochondria [38], suggesting that ROS generation might be associated with respiration. Roles of ROS derived from the mitochondria as signaling molecule have not been elucidated; however, the significance of RBOH-dependent ROS in the regulation of pollen tube elongation has been evidenced. RESPIRATORY BURST OXIDASE HOMOLOG H and J were shown to be essential for the maintenance of pollen tube integrity in the TT of pistil. The pollen tubes of double mutant *rbohh/rbohj* exhibited bursting in vitro and inhibited growth in pistils [7,8]. In addition, pollen tube integrity during elongation was also found to be controlled by receptor complex composed of ANXUR1/2 (ANX1/2), BUDDHA’S PAPER SEAL 1/2 (BUPS1/2), and LORELEI-LIKE-GPI-ANCHORED PROTEIN 2/3 (LLG2/3), which perceives autocrine peptide ligands RALF4/19 [103,104,105,106,107]. This receptor complex functions upstream to RBOHH and RBOHJ during pollen tube elongation in the pistil [7,108]. The activation of RBOHs is mediated by GUANINE NUCLEOTIDE EXCHANGE FACTOR (GEFs) and RHO of PLANTS (ROPs) in the downstream of ANX1/2 [106,109]. Significance of ANX1/2-BUPS1/2-LLG2/3 complex in the maintenance of pollen tube integrity via ROS production can be also supported by the fact that an Arabidopsis *llg2/3* double mutant showed reduced ROS accumulation in pollen tubes as well as burst of tips in vitro [106]. Furthermore, functions of LLG2/3 and ROS are associated with synthesis of cell wall components of pollen tube [110,111]. RAPID ALKALINIZATION FACTOR 4 was shown to alter the composition of the pollen tube wall, such as callose and pectin, which are correlated with the pollen tube integrity and elongation [105,106]. Pollen tubes of *llg2/3* double mutant that accumulates reduced level of ROS also exhibited altered pectin and callose deposition at the tip wall of the pollen tube [106]. Although findings described above clearly suggest that pollen tube elongation is regulated by energy generation via respiratory pathways together with ROS signals and carbohydrate metabolisms (Figure 3), the mode of integration between these mechanisms has still not been elucidated. It is necessary to analyze the effects of the impairment in respiratory pathways on the mechanisms involving the receptor complex and ROS, or vice versa.

Several mechanisms required for the guidance of pollen tube elongation have been proposed. Although the attraction from the pistil is not required for the elongation of pollen tube in the style, pollen tube elongation depends on the elongation ability of the pollen tube itself [112]. On the other hand, attraction from the pistil is essential for pollen tube elongation in the TT. Arabidopsis possesses approximately 60 ovules per ovule [113], whereas more than 100 pollen tubes grow in the pistil [114]. However, only one pollen tube can reach to one ovule, and the probability that multiple pollen tubes invade one ovule and fertilize is 1% in Arabidopsis [115]. In previous studies, several AGPs were implicated in the control of the guidance of pollen tube elongation. In tobacco, TTS proteins, AGPs that provide nutrients (arabinogalactans) to pollen tubes from the TT as mentioned above [98], were also shown to be involved in the guidance of pollen tube tip to ovules. TTS might function as adhesion molecules that interact with pollen tube [116]. In addition, an AGP, ARABINOXYLAN PECTIN ARABINOGALACTAN PROTEIN 1 (APAP1), was shown to crosslink with pectin and hemicellulose polysaccharides in cell walls, forming a continuous network between polysaccharides and cell wall proteins, which might be required for pollen tube guidance [117,118]. In Arabidopsis, AGP1, AGP12, and AGP15 were found to be expressed in the funiculus [116]. It was proposed that these AGPs might be important in the step in which pollen tubes from the TT grow along the funiculus. Several proteins of the CNGC family were also suggested to be required for pollen tube elongation and guidance [119,120]. For example, Arabidopsis mutant deficient in CNGC18 showed abnormality of pollen tube guidance as well as branching of pollen tubes [120,121]. The double mutant of Arabidopsis deficient in CNGC7 and CNGC8 (*cngc7/cngc8*) demonstrated bursting of pollen tubes as well as sterility [119]. The phenotypes of *cngc7/cngc8* were similar to that of *rbohh/rbohj* double mutant [108], indicating the integration of functions of CNGCs with RBOH-dependent ROS production in pollen tubes. Integration between ROS-CNGC signals with functions of AGPs in pollen tube elongation and guidance has not been evidenced yet. However, we should not ignore such possibility because the link between RBOHs and AGP2 was proposed in the tapetum [40]. These mechanisms to guide pollen tube growth might be tightly linked to the processes in which female gametophytes perceive pollen tubes (see below).

## 6. Pollen Tube Perception by the Female Gametophyte and Tube Rapture

In hitherto studies, several key players involved in pollen tube perception by female gametophyte have been identified. In torenia (*T. fournieri*), the terminal residue of arabinogalactan polysaccharide (AMOR) was identified at the stigma [39]. AMOR induces the ability of pollen tubes to react with a peptide called LURE, which is essential in the final stages of guidance to the pollen tube pit. The attracted and elongated pollen tube passes the TT and reaches to the ovule pit. LURE is required for guidance of the pollen tube that just has passed through the TT. Although effective range of LURE is approximately 20 µm, the distance between the TT and the micropyle is about 100 µm. Thus, before LURE acts, it is possible that other factors might contribute to the guidance of pollen tube tip to ovule [122]. Furthermore, ethylene-independent signaling controlled by ethylene precursor 1-aminocyclopropane-1-carboxylic acid (ACC) was recently shown to regulate pollen tube attraction toward the ovule via promoting LURE1.2 secretion and cytosolic Ca^2+^ elevation [123].

When pollen attaches to the stigma, even before pollen tubes reach to the ovules, increase in H_2_O_2_ accumulation was detected in synergids [64]. Application of ROS scavengers or inhibitors resulted in defects of pollen tube perception by ovules. Although several pollen tubes reached to the ovules even in the presence of ROS scavengers, these tubes failed to burst and continued to grow in the synergids [124]. These findings suggest that ROS accumulation in synergids is essential for the proper guidance and bursting of pollen tube tip. In addition, it was demonstrated that ROS generated via functions of RBOH in the female gametophyte is required for the pollen tube rupture in the synergid [124]. FERONIA that is involved in pollen hydration (see above) was also found to mediate pollen tube rupture by inducing RBOHD-dependent ROS production via the functions of GEFs and ROPs [125,126]. GUANINE NUCLEOTIDE EXCHANGE FACTORs activate ROPs to stimulate RBOH-dependent ROS production. In addition, glycosylphosphatidyninositol-anchored protein LORELEI (LRE) and early nodulin-like protein (ENODLs) were shown to be co-receptors of FER signals involved in ROS production in the synergid [124,125]. Significance of LRE in the regulation of FER signal was also supported by the finding that plants deficient in LRE exhibited reduced ROS in ovules accompanied by pollen tube overgrowth [124]. RAPID ALKALINIZATION FACTOR 34, expressed in mature ovules, functions antagonistically with RALF4/19, which are known to be positive regulators of pollen tube integrity. RAPID ALKALINIZATION FACTOR 34 competes with RALF4/19 for binding to BUPs and ANXs and induce bursting of pollen tubes. Thus, RALF34 disrupts RALF4/19-dependent pollen tube integrity and allows pollen tubes to respond to signals in synergid mediated by FER [103,127]. Furthermore, Ca^2+^ also functions as key regulator of pollen tube burst in synergids. Indeed, pollen tube burst mediated by FER is dependent on high level of Ca^2+^ channel activity that leads high Ca^2+^ accumulation in the synergids [124]. MILDEW RESISTANCE LOCUS O (MLOs) expressed in the synergids was also shown to be linked to ROS regulatory systems in the synergids [3]. MLOs interact with CNGC18 to control Ca^2+^ gradient in the pollen tubes, leading to the acceleration of their growth [128]. In addition, MLO7 accumulates in the Golgi during synergid differentiation and is localized in the filiform apparatus during pollen tube perception [129]. This pattern of MLO7 accumulation could be associated with a possible role in the regulation of synergid response to ROS accumulation.

Perception of pollen tube tips by female gametophyte and following pollen tube rapture are sequential events that might be tightly linked to achieve proper fertilization. We should not therefore discard the possibility that mechanisms regulated by AMOR and LURE could trigger ROS-dependent pollen tube rapture in the synergids. Under the current situation, it is urgent to find key players other than AMOR and LURE that guide the pollen tube tip to the micropyle.

## 7. Conclusions

Overall, numerous findings discussed above suggest that temporal-spatial coordination between ROS regulatory systems and carbohydrate transports and metabolisms is essential for the various processes underlying reproductive development. Thus, timing and intensity of signals that function in different reproductive tissues need to be fine-tuned, and different signals should be integrated. We can propose several key processes that integrate ROS regulatory systems and carbohydrate transport and metabolisms. ROS-dependent PCD should be one of these key processes that might be tightly linked to carbohydrate transports and metabolisms. For example, PCD in the tapetum is essential for the nutrient supply to the developing pollen via function of sugar transporters [6,26,32]. In addition, PCD in the TT might be also associated with the carbohydrate supply for the pollen tube elongation and guidance [92,93,94,95]. Mitochondrial functions are also important to link ROS regulatory systems and carbohydrate metabolisms. Mitochondria is well known as a major source of ROS. Thus, it is possible that mechanisms to utilize ROS generated via active respiration have been evolved in plants. Indeed, strict regulation of mitochondrial ROS was shown to be essential for development of female gametophytes [63,64]. Furthermore, distribution of ROS accumulation in pollen tube coincided with that of mitochondria [38]. It is necessary to uncover functions of mitochondrial ROS in the regulation of pollen tube elongation. As well as their role in ROS production, mitochondria are also tightly linked to the carbohydrate metabolic pathways, such as glycolysis and TCA cycle, that are essential for the energy supply to pollen tubes [38]. It is likely that mitochondria function as a hub to modulate ROS signals and carbohydrate metabolisms. Moreover, we should not ignore the significance of AGPs in the regulation of reproductive development. Arabinogalactan proteins were shown to be distributed through the reproductive organs in plants, and their contributions to broad range of processes in reproductive tissues have been suggested [42,116]. Interestingly, integration of AGP with ROS-dependent PCD in the tapetum has been proposed [53]. However, our understanding associated with the functions of AGPs in reproductive development is still poor. Thus, it is necessary to further elucidate the links between ROS signals and AGP functions in future studies. In this review, we summarized the mechanisms underlying different steps of reproductive development. However, it is almost impossible to completely separate these sequential events. Thus, it should be noted that mechanisms regulating the certain processes can dramatically affect the following processes.

The information presented in this review could be applied for the establishment of strategies to increase yield of grains and legumes. For example, genetic engineering focusing on key regulators of mechanisms discussed in this review might result in efficient control of ROS or carbohydrate signals, leading to improvement of yields. In addition, for the countries in which cultivation of genetically modified crops is highly restricted or prohibited, biostimulants or compounds that control important metabolisms could be found on the basis of the information of signaling networks. We hope this review will be useful for breeders or researchers of agriculture.

## Data Availability

No new data were created or analyzed in this study. Data sharing is not applicable to this article.
